# Clinical and immunological characteristics of *Aspergillus fumigatus*-sensitized asthma and allergic bronchopulmonary aspergillosis

**DOI:** 10.3389/fimmu.2022.939127

**Published:** 2022-08-02

**Authors:** Hao Chen, Xinyu Zhang, Li Zhu, Nairui An, Qing Jiang, Yaqi Yang, Dongxia Ma, Lin Yang, Rongfei Zhu

**Affiliations:** ^1^ Department of Allergy, Tongji Hospital, Tongji Medical College, Huazhong University of Science and Technology, Wuhan, China; ^2^ Department of Allergy and Clinical Immunology, Guangzhou Institute of Respiratory Health, State Key Laboratory of Respiratory Disease, National Clinical Research Center of Respiratory Disease, First Affiliated Hospital of Guangzhou Medical University, Guangzhou, China; ^3^ Department of Hematology, Tongji Hospital, Tongji Medical College, Huazhong University of Science and Technology, Wuhan, China

**Keywords:** *Aspergillus fumigatus*, asthma, allergic bronchopulmonary aspergillosis, CD4^+^T cell, IgE

## Abstract

**Background:**

*Aspergillus fumigatus* (*A.f*) is a common airborne allergen that contributes to allergic asthma. In some patients, *A.f* can colonize in the airway and lead to allergic bronchopulmonary aspergillosis (ABPA). However, our understanding of the pathogenesis of *A.f*-sensitized asthma and ABPA remains inadequate.

**Objective:**

We aimed to investigate the clinical and immunological characteristics of *A.f*-sensitized asthma and ABPA.

**Methods:**

A total of 64 ABPA and 57 A*.f*-sensitized asthma patients were enrolled in the study, and 33 non-*A.f*-sensitized asthma patients served as the control group. The clinical and immunological parameters included lung function, fractional exhaled nitric oxide (FeNO), induced sputum and blood cell analysis, specific IgE/IgG/IgA of A.f and its components, cytokines (IL-33, IL-25, and TSLP) and CD4^+^T cell subsets.

**Results:**

The eosinophils in blood, induced sputum, and FeNO were significantly higher in ABPA patients compared to that in *A.f*-sensitized patients. The combination of FeNO and eosinophils (EO) parameters presented good diagnostic efficiency in differentiating *A.f* (+) asthma from ABPA, with a sensitivity of 80% and a specificity of 100%. Specific IgE, IgG, and IgA against *A.f* also increased in ABPA patients. However, serum IL-25, IL-33, and TSLP showed no significant differences between the two groups. Cell analysis showed an increase in IFN-γ^+^Th1 cells in the ABPA patients. FlowSOM analysis further confirmed that the frequency of CD3^+^CD4^+^PD-1^+^CD127^+^IFN-γ^+^T cells was higher in ABPA patients.

**Conclusion:**

Our findings suggest the distinct humoral and cell immunological responses in *A.f*-sensitized asthma and ABPA patients. ABPA patients have more severe eosinophilic inflammation and enhanced Th1 responses compared with *A.f*-sensitized asthma patients.

## Introduction

Asthma is a heterogeneous disease characterized by chronic airway inflammation and airway hyperresponsiveness. The main clinical manifestations are recurrent wheezing, shortness of breath, chest tightness, or cough (1). According to recent epidemiological surveys in China (2), more than 45 million adults over the age of 20 suffered from asthma, and the majority of them suffered from allergic asthma. Fungi, especially *Aspergillus fumigatus* (*A.f*), are common airborne allergens that cause allergic asthma (3). The colonies of *A.f* are fluffy or flocculated and can grow rapidly. With thermophilic feature, *A.f* can colonize in the airway and cause invasive infection; its sensitization is also a risk factor for severe asthma (4).

Allergic bronchopulmonary aspergillosis (ABPA) is a chronic inflammation triggered by repeated exposure to *A.f*, which has colonized for a long time in the airway (5). The clinical manifestations include chronic asthma or bronchiectasis and recurrent wandering pulmonary shadows or mucus blockage in lung images (6). While ABPA was mostly found in patients with bronchial asthma or cystic fibrosis in Western countries, it was more common in patients with asthma and bronchiectasis in China (7). A study revealed that the estimated prevalence of ABPA in adults with asthma was at 2.5% (8) and could rise up to 45% in patients with *A.f*-sensitized asthma (9). As the symptoms were extremely similar to those of asthma and other airway diseases, ABPA was always misdiagnosed in clinical practice. A study in China revealed that nearly 70% of the ABPA patients in the country had been misdiagnosed. Among them, 21% were misdiagnosed as tuberculosis and received anti-tuberculosis therapy for at least 1 year. Some patients would receive high-dosage inhalant or systemic steroids and antibiotics but were always resistant to the treatments (10). Thus, it is of great significance to distinguish ABPA from asthma, especially *A.f*-sensitized asthma.

Currently, the diagnosis for ABPA mainly relies on total IgE (tIgE), specific IgE (sIgE) against *A.f*, pulmonary imaging, and blood eosinophil count. However, most of these indicators are non-specific and not very sensitive. A detailed mechanism and surrogate indicators of ABPA and *A.f*-sensitized asthma still need further investigation. In this study, we investigated clinical and immunological characteristics of ABPA and *A.f*-sensitized asthma with a view of elucidating the underlying mechanism and exploring potential biomarkers to discriminate ABPA from *A.f*-sensitized asthma.

## Materials and methods

### Patients

This study was conducted from September 2018 to June 2021 in the Department of Respiratory Medicine at the First Affiliated Hospital of Guangzhou Medical University and the Department of Allergy, Tongji Hospital, Tongji Medical College. The inclusion criteria for patients enrolled in our study were those who were diagnosed with asthma or ABPA, based on Global Initiative for Asthma (GINA) guidelines (http://ginasthma.org/) and ABPA criteria (11). The exclusion criteria were (1) patients with chronic diseases such as tuberculosis, chronic obstructive pulmonary disease, coronary heart disease, hypertension, diabetes, and tumor and (2) patients who received systemic glucocorticoids or monoclonal antibodies within 1 month that might affect the levels of eosinophil or tIgE before enrollment. The study was approved by the Independent Ethical Committee of First Affiliated Hospital of Guangzhou Medical University (No. GYFYY-2016-73) and Tongji Hospital (No. TJ-IRB20210761). Each participant or their guardian provided a written informed consent.

Based on clinical data, sIgE against *A.f*, chest computerized tomography (CT) reports, and peripheral blood eosinophil counts, the enrolled patients were divided into three groups: non-*A.f*-sensitized asthma [*A.f* (−) asthma], *A.f*-sensitized asthma [*A.f* (+) asthma], and ABPA. The demographic data included gender, age, height, weight, and other basic information of the patients. The count and percentage of peripheral blood cells including white blood cell (WBC), neutrophils (NEUT), lymphocytes (LY), and eosinophils (EO) and infection-related indicators such as erythrocyte sedimentation rate (ESR) and procalcitonin (PCT) were also collected in the first detection after hospitalization. According to the GINA guideline, asthma control test (ACT) was used for each patient in the enrollment stage to evaluate the control level of these three groups. The total score of ACT was 25, in which ≥20 was defined as controlled and <20 was uncontrolled.

### Clinic and immunological parameters

#### Lung function and fractional exhaled nitric oxide

Lung function was measured by spirometry on a MasterScreen Pneumo (Jaeger, Wurzburg, Germany) spirometer. The parameters for such measurement included forced vital capacity (FVC), forced expiratory volume in 1 s (FEV1), FEV1/FVC, peak expiratory flow (PEF), maximum mid-expiratory flow (MMEF), maximal expiratory flow 50 (MEF50), and maximal expiratory flow 25 (MEF25). Fractional exhaled nitric oxide (FeNO) level was measured with a portable electrochemical device (NIOX MINO; Aerocrine AB, Stockholm, Sweden), and the upper normal limit was 25 ppb.

#### Induced sputum cytology classification

After gargling with water, the subjects underwent sputum induction by inhaling 3% sodium chloride solution with ultrasonic atomization for 15 min and coughed up the sputum to the Petri dish. Sputum of 3–5 ml was weighed and added into 0.1% dithionitol solution with four times volume. After 1 min of vortex shock, the sputum was incubated in a 37°C water bath for 10 min until the specimen was liquefied. The samples were filtered by a 300-mesh nylon filter, and the filtrate was centrifuged at 3,000 rpm for 10 min. The cell precipitate was prepared into cell smears, which were dried in a 55°C oven. The cell smears were fixed with 10% formaldehyde and stained with Raysgiemsa for 15–20 min. Sputum cells including eosinophils (SEO), neutrophils (SNEUT), macrophages (SMø), and lymphocytes (SLY) were classified and counted under a microscope.

#### IgE, IgG, and IgA detection

The serum sample was collected to analyze tIgE and *A.f*-sIgE using ImmunoCAP 1000 provided by Thermo Fisher Scientific Inc. Positive sIgE was categorized into six classes: class 1 (≥0.35–<0.70 KU/L), class 2 (≥0.70–<3.50 KU/L), class 3 (≥3.50–<17.50 KU/L), class 4 (≥17.50–<50 KU/L), class 5 (≥50–<100 KU/L), and class 6 (≥100 KU/L).

Specific IgA, IgG4, and IgE against *A.f* and its components (Asp f1, Asp f3, and Asp f9) were measured by *A.f* components test kit (Hangzhou Zheda Dixun Biological Gene Engineering Co, Ltd, Hangzhou, China). The recombinant *A.f* and its components (Asp f1, Asp f3, and Asp f9) were precoated on the chip. After serum was incubated on the chip for 1 h, anti-human IgE/IgA/IgG4 antibodies (conjugated by biotin) and alkaline phosphatase–streptavidin were added consecutively. The concentrations of IgE/IgA/IgG4 against *A.f* were calculated by a series of protein standardization. The positive cutoff value of sIgG4 was set by 95% percentile of upper limit of 210 healthy non-allergic donors; sIgG4 above 156 U_A_/ml, sIgA above 10 U/ml, and sIgE above 0.35 IU/ml were defined as positive.

### Interleukin-33, IL-25, thymic stromal lymphopoietin assay

Concentrations of interleukin (IL)-33 (EHC151, Neobioscience, China), IL-25 (EHC180, Neobioscience, China), and TSLP (EK0958, BOSTER, China) in serum were determined using their specific enzyme-linked immunosorbent assay (ELISA) kits according to the manufacturer’s instructions.

#### Cell staining and flow cytometric analysis

Peripheral blood mononuclear cells (PBMCs) of patients were isolated and stored in liquid nitrogen. Thawed PBMCs of ABPA patients and *A.f* (+) asthma patients were incubated with 25 µg/ml *A.f* allergen extracts and 100 ng/ml PMA (phorbol-12-myristate 13-acetate, MCE, China) for 6 h. PBMCs of house dust mite (HDM)-positive group were incubated with 25 µg/ml HDM and 100 ng/ml PMA also for 6 h as control, and then, the harvested cells were prepared at a concentration of 1×10^6^ in 100 µl flow cytometry (FACS) staining buffer and were stained with Live/Dead Fixable Read cell stain kit (Invitrogen, USA), anti-human CD3, CD4, CD183 (CXCR3), CD294 (CRTH2), CD25, CD127, IL-4, IL-13, and IFN-γ antibodies (Biolegend, USA), and CD279 (PD-1), IL-10, and CD185 (CXCR5) (BD Biosciences, USA). T subsets were gated by Th1 (CD3^+^CD4^+^CXCR3^+^), Th2(CD3^+^CD4^+^CRTH2^+^), Tfh (CD3^+^CD4^+^CXCR5^+^PD-1^+^), and Treg (CD3^+^CD4^+^CD25^+^CD127^low/−^) (12, 13). Stained cells were acquired with FACS Caliber (BD, Biosciences, Milpitas, CA, USA), and the data were analyzed with FlowJo software.

#### FlowSOM algorithm

The FlowSOM algorithm helps to obtain an overview of all markers and expression on all cells to identify novel subsets. Viable cells were downsampled to 1,000 cells per triplicate and donor using the Downsample version 3.3 plugin for FlowJo and were concatenated to one sample per group. FlowSOM was performed for each study group separately. Flow cytometry standard (FCS) files were manually pregated as CD3^+^CD4^+^ Live/Dead and were sorted into 15 metaclusters, which provided sufficient metaclusters to capture the expected number of unique cell types while potentially uncovering other biologically interesting populations. For the second study group, we chose the option “apply on map” of the first analyzed study group. Heat maps were generated for both study groups including all parameters. The resulting metaclusters were manually inspected for the expression of marker using the heat map.

### Statistical analysis

Continuous variables with normal distribution were represented as mean ± standard deviation (SD), while non-normal distribution was represented by median and range interquartile. Quantitative data among multiple groups was analyzed by ANOVA and Kruskal–Wallis tests. Categorical variables in terms of frequency and percentage were expressed and were compared using the χ^2^ or Fisher’s exact tests as appropriate. Spearman rank test was used for correlation evaluation. Multivariate logistic regression models were constructed, and least absolute shrinkage and selection operator (LASSO) regression method was used for feature selection, and the area under receiver operating characteristic (ROC) curve (AUC) was used to verify the sensitivity and specificity of models. Statistical analyses were performed using SPSS version 20.0 (IBM, Chicago, IL) and R package version 3.5.1. p<0.05 was considered as statistically significant.

## Results

### Patient characteristics

A total of 154 patients were recruited and divided into three groups, including 33 patients with *A.f* (−) asthma, 57 patients with *A.f* (+) asthma, and 64 ABPA patients. There was a significant difference in age between ABPA and *A.f* (+) asthma (p<0.05). The parameters of lung function showed that FVC (%), FEV1 (%), FEV1/FVC (%), PEF (%), MEF75/25 (%), MEF50 (%), and MEF25 (%) in the ABPA group and the *A.f* (+) asthma group were lower than those in the *A.f* (−) asthma group (both p<0.05), but these parameters showed no statistical significance between the ABPA and *A.f* (+) asthma groups. FeNO in the ABPA group was higher than that in the *A.f* (+) asthma and the *A.f* (−) asthma group (both p<0.01). In venous blood cell analysis, both EO and EO% in ABPA group were higher than those in *A.f* (+) asthma and *A.f* (−) asthma group (p<0.001). In the induced sputum cytology classification, the SEO% of the ABPA group was higher than that of *A.f* (+) asthma and *A.f* (−) asthma groups (p<0.05), and SNEUY% and SMø% in the ABPA group presented significant difference compared with *A.f* (−) asthma group (both p<0.05) (**Table 1**).

**Table 1:** The comparison of clinical parameters in the three groups.

	**^a^ABPA (n=64)**	**^b^ *A.f* (+) Asthma (n=57)**	**^c^ *A.f* (−) Asthma (n=33)**	**Comparison (p-value)**		
				***a vs*. *b***	***a vs*. *c***	***b vs*. *c***
**Age (years)**	45.0 (33.5–52.8)	33.0 (14.5–52.0)	38.0 (9.0–61.5)	**0.026**	0.161	0.703
**Male/Female**	40/24	34/23	20/13	0.926	0.924	0.772
BMI (kg/m²)	20.73 (18.64–24.29)	21.64 (18.36–24.80)	20.98 (16.78–25.50)	0.830	0.827	0.976
Lung function						
FVC (%)	79.87 ± 16.97	83.19 ± 19.07	94.73 ± 15.98	0.649	**0.000**	**0.007**
FEV1 (%)	64.35 (46.25–84.32)	60.20 (41.00–90.50)	88.90 (76.65–102.20)	0.919	**0.000**	**0.001**
FEV1/FVC (%)	78.34 ± 20.76	75.46 ± 18.70	90.39 ± 11.17	0.638	**0.005**	**0.001**
PEF (%)	64.28 ± 25.39	62.65 ± 26.25	85.80 ± 19.55	0.656	**0.000**	**0.000**
MMEF75/25 (%)	33.08 (12.80–57.83)	22.00 (14.50–60.60)	55.10 (41.36–76.15)	0.379	**0.006**	**0.001**
MEF50 (%)	37.05 (15.40–63.25)	22.30 (15.00–68.20)	54.41 (44.05–75.05)	0.258	**0.009**	**0.000**
MEF25 (%)	29.45 (13.67–54.80)	24.60 (10.20–53.42)	48.50 (32.25–69.23)	0.425	**0.021**	**0.003**
FeNO	76.00 (52.00–89.50)	40.00 (32.00–52.00)	44.00 (23.50–69.00)	**0.000**	**0.001**	0.782
Venous blood cell analysis						
WBC (10^9/L)	8.00 (6.74–9.86)	8.52 (6.93–9.90)	8.50 (6.20–10.02)	0.882	0.979	0.745
NEUT%	58.61 ± 13.30	59.40 ± 15.35	58.16 ± 14.79	0.581	0.856	0.659
LY%	22.90 (18.03–35.00)	28.40 (18.40–34.00)	27.10 (20.40–41.40)	0.709	0.134	0.401
EO%	10.60 (7.20–12.55)	4.10 (1.20–7.30)	4.30 (1.20–7.15)	**0.000**	**0.000**	0.917
NEUT (10^9/L)	4.25 (3.35–6.28)	4.50 (3.30–6.40)	4.40 (3.60–6.25)	0.716	0.898	0.859
LY (10^9/L)	1.80 (1.48–2.53)	2.40 (1.50–2.60)	2.10 (1.55–3.05)	0.528	0.293	0.735
EO (10^9/L)	1.65 (1.09–2.26)	0.33 (0.10–0.54)	0.27 (0.09–0.65)	**0.000**	**0.000**	0.606
Induced sputum cytology classification						
SNEUT%	71.95 ± 20.41	60.39 ± 24.92	54.24 ± 25.66	0.067	**0.003**	0.261
SMø%	5.00 (0.50–20.30)	9.50 (1.00–25.00)	16.00 (4.63–44.59)	0.367	**0.024**	0.135
SEO%	21.15 (7.60–35.75)	5.55 (2.50–14.50)	5.85 (2.75–9.50)	**0.000**	**0.000**	0.681
SLY%	1.02 (0.50–2.00)	1.05 (0.50–1.50)	1.25 (1.00–2.50)	0.844	0.146	0.116
Infection index						
ESR (mm/h)	18.00 (8.50–26.25)	12.00 (7.00–20.00)	12.00 (10.00–21.50)	0.320	0.473	0.623
PCT (positive rate)	80.95%	77.14%	75.76%	0.682	0.586	0.893

ABPA, allergic bronchopulmonary aspergillosis; A.f (+) asthma, Aspergillus fumigatus-sensitized asthma; A.f (−) asthma, non-Aspergillus fumigatus-sensitized asthma; BMI, body mass index; FVC, forced vital capacity; FEV1, forced expiratory volume in 1 s; PEF, peak expiratory flow; MMEF, maximum mid-expiratory flow; MEF50, maximal expiratory flow 50; MEF25, maximal expiratory flow 25; FeNO, fractional exhaled nitric oxide; SNEUT, sputum neutrophils; SMø, sputum macrophages; SEO, sputum eosinophils; SLY, sputum lymphocytes; WBC, white blood cell; NEUT, neutrophils; LY, lymphocytes; EO, eosinophils; ESR, erythrocyte sedimentation rate; PCT, procalcitonin. Bold font indicates statistical significance (p<0.05).

**Figure 1:**
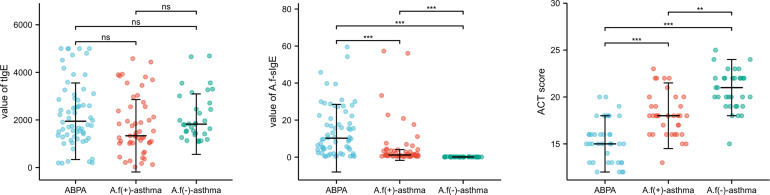
The comparison of tIgE, *A.f*-sIgE, and ACT score in three groups. ABPA, allergic bronchopulmonary aspergillosis; *A.f* (+) asthma, *Aspergillus fumigatus*-sensitized asthma; *A.f* (−) asthma, non-*Aspergillus fumigatus* sensitized asthma. ns, no significance; **p<0.01; ***p<0.001.

There was no difference in tIgE, but significant difference in *A.f*-sIgE among the three groups, in which the ABPA group had significantly higher sIgE level than the other two groups (10.24 *vs*. 1.15 *vs*. 0.07, p<0.05). ACT scores were statistically different (15 *vs*. 18 *vs*. 21, p<0.01) among the three groups, and the lowest was found in the ABPA group (**Figure 1**).

**Figure 2:**
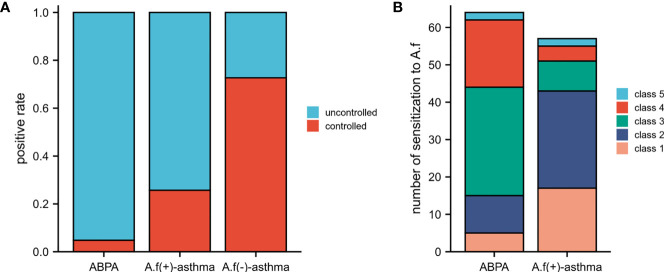
Control situation and class of *A.f*-sIgE in different groups. **(A)** Control situation among ABPA, *A.f* (+) asthma, and *A.f* (−) asthma. **(B)** Class of *A.f*-sIgE between ABPA and *A.f* (+) asthma. ABPA, allergic bronchopulmonary aspergillosis; *A.f* (+) asthma, *Aspergillus fumigatus*-sensitized asthma; *A.f* (−) asthma, non-*Aspergillus fumigatus*-sensitized asthma.

It should be noted that 95.20% of ABPA patients did not reach the control level; the percentage was 74.30% in *A.f* (+)-asthma patients and only 27.30% in *A.f* (−)-asthma patients (**Figure 2A**). The level of *A.f*-sIgE in 76.2% of the ABPA patients was classified as high concentration (classes 3−6), while only 25.7% of the *A.f* (+) asthma patients had sIgE level equal or above class 3 (**Figure 2B**).

### Correlation of clinical and immunological parameters in different groups

**Figure 3:**
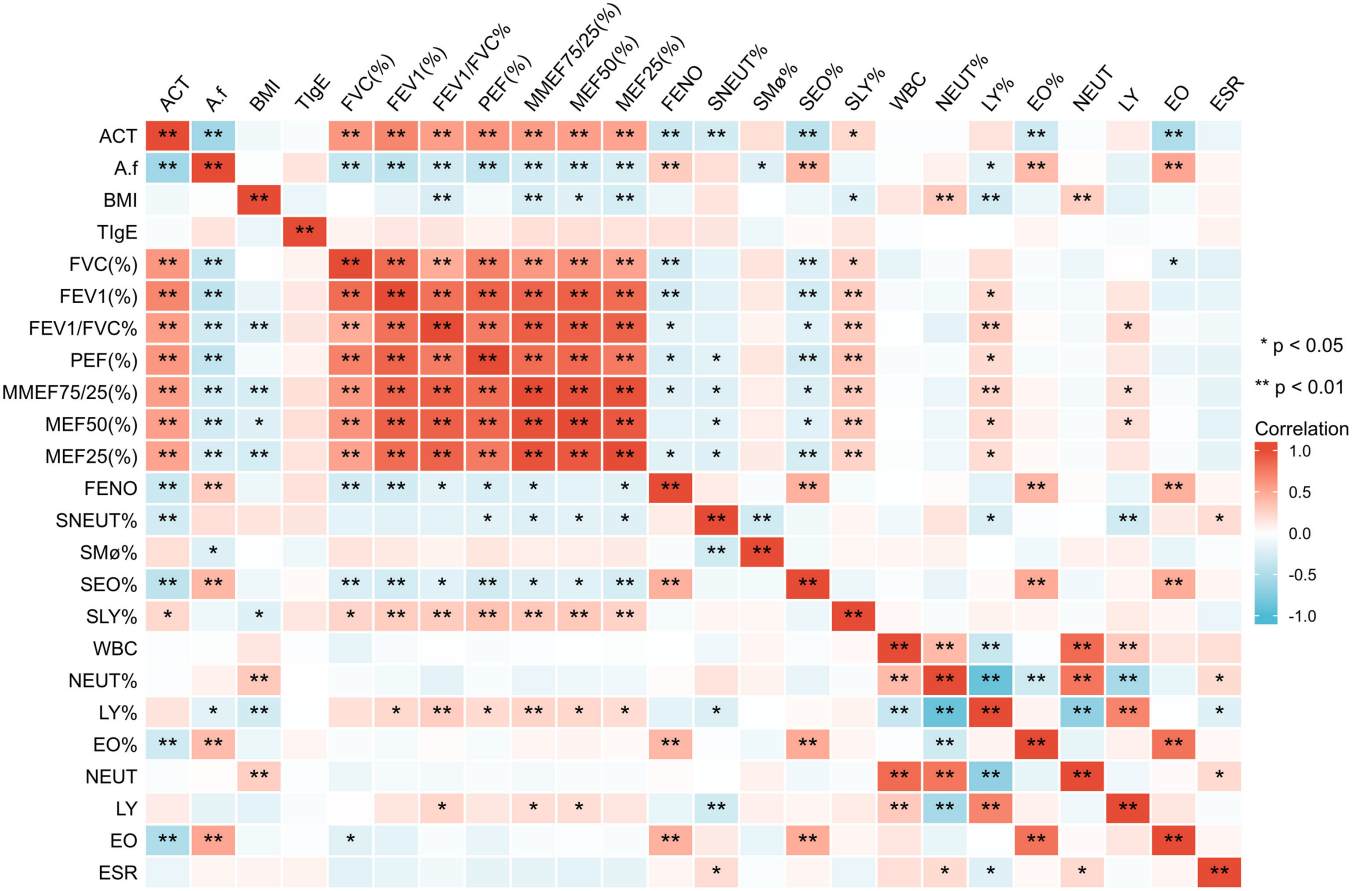
Correlation of clinical parameters in all the patients. ACT, asthma control test; *A.f*, *Aspergillus fumigatus*; BMI, body mass index; tIgE, total IgE; FVC, forced vital capacity; FEV1, forced expiratory volume in 1 s; PEF, peak expiratory flow; MMEF, maximum mid-expiratory flow; MEF50, maximal expiratory flow 50; MEF25, maximal expiratory flow 25; FeNO, fractional exhaled nitric oxide; SNEUT, sputum neutrophils; SMø, sputum macrophages; SEO, sputum eosinophils; SLY, sputum lymphocytes; WBC, white blood cell; NEUT, neutrophils; LY, lymphocytes; EO, eosinophils; ESR, erythrocyte sedimentation rate; PCT, procalcitonin. *p<0.05; **p<0.01.

There were positive correlations between ACT score and all lung function parameters (p<0.01) and negative correlations between ACT and *A.f*-sIgE, FeNO, SEO%, EO%, and EO (p<0.01). *A.f*-sIgE was positively correlated with FeNO value, SEO%, EO%, and EO (p<0.01) and negatively correlated with ACT score and all lung function parameters (p<0.01). All lung function parameters except MEF50 (%) were negatively correlated with FeNO and SEO% (p<0.05). FeNO value was positively correlated with SEO%, EO%, and EO (p<0.01) (**Figure 3**).

**Figure 4:**
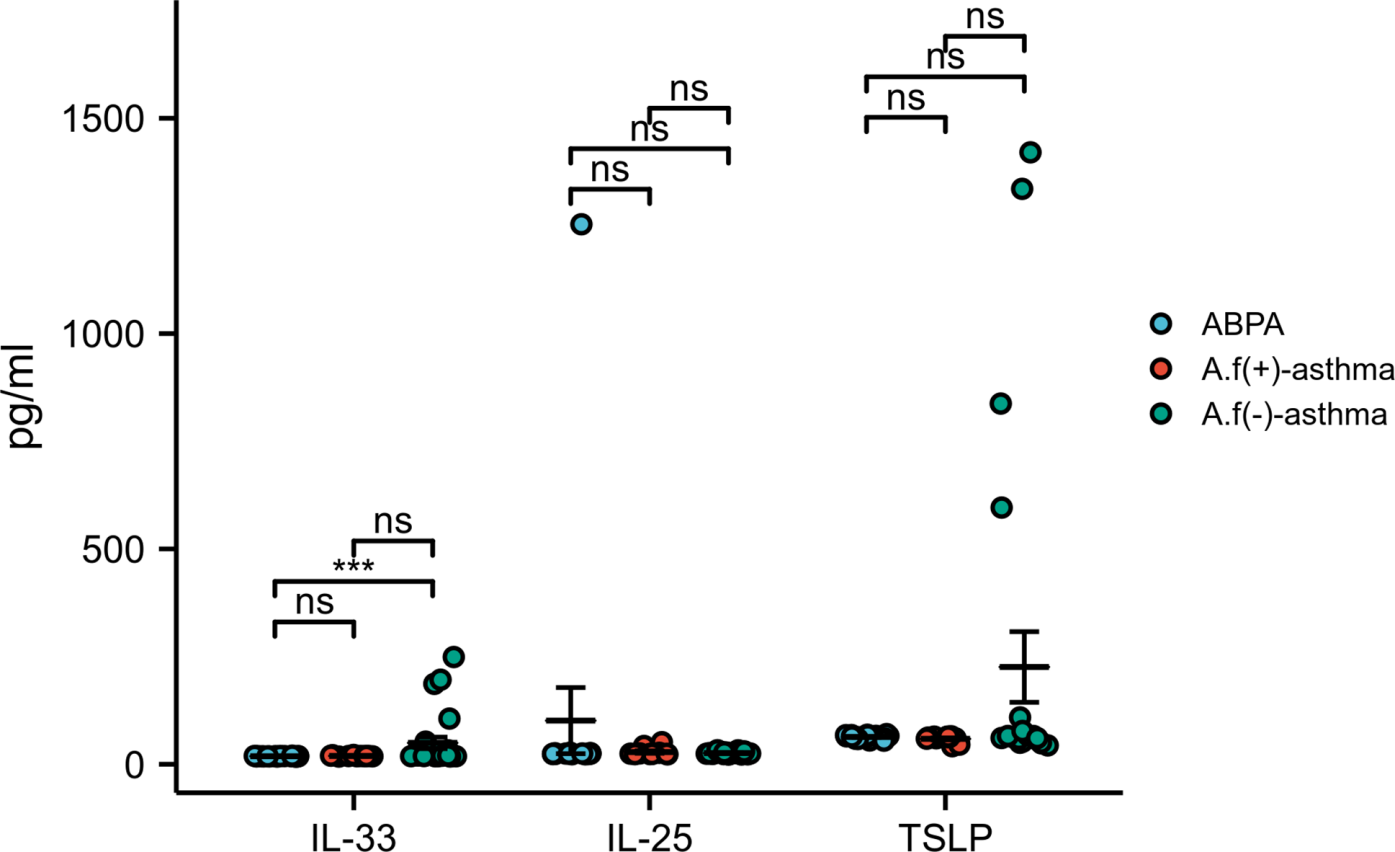
Correlation of clinical parameters in the three groups. ACT, asthma control test; *A.f*, *Aspergillus fumigatus*; BMI, body mass index; tIgE, total IgE; FVC, forced vital capacity; FEV1, forced expiratory volume in 1 s; PEF, peak expiratory flow; MMEF, maximum mid-expiratory flow; MEF50:maximal expiratory flow 50; MEF25, maximal expiratory flow 25; FeNO, fractional exhaled nitric oxide; SNEUT, sputum neutrophils; SMø, sputum macrophages; SEO, sputum eosinophils; SLY, sputum lymphocytes; WBC, white blood cell; NEUT, neutrophils; LY, lymphocytes; EO, eosinophils; ESR, erythrocyte sedimentation rate; PCT, procalcitonin. ***p < 0.001; ns indicates no statistical significance.

For the ABPA group, ACT was positively correlated with all lung function parameters (p<0.05), *A.f*-sIgE value was positively correlated with tIgE (p<0.05), and tIgE value was negatively correlated with EO (p<0.05). For the *A.f* (+)-asthma group, ACT score was positively correlated with all lung function parameters (p<0.01); all the lung function parameters except FEV1 (%) were negatively correlated with SEO% (p<0.05). FeNO was positively correlated with SEO% (p<0.01). For the *A.f* (−)-asthma group, ACT value was positively correlated with FEV1 (%), FEV1/FVC(%), MEF75/25(%), MEF50 (%), and MEF25 (%) (p<0.05); A.f-sIgE was negatively correlated with MEF50 (%) (p<0.05); FeNO was positively correlated with SEO% and EO (p<0.05); and FVC (%) and MEF50 (%) were positively correlated with EO% (p<0.05) (**Figure 4**).

### Predictive value of clinical parameters in *A.f* (+) asthma and ABPA

**Figure 5:**
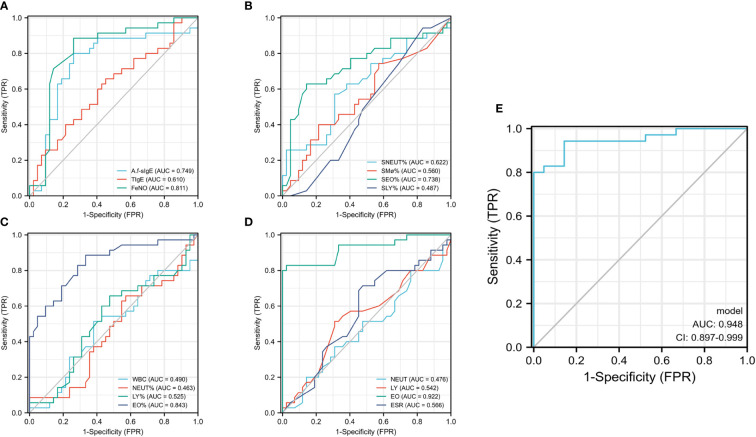
Predictive value of clinical parameters in *A.f* (+) asthma and ABPA. *A.f* (+) asthma, *Aspergillus fumigatus*-sensitized asthma; ABPA, allergic bronchopulmonary aspergillosis. **(A–D)** ROC curve analyzed the predictive value of clinical parameters for *A.f* (+) asthma and ABPA; **(E)** ROC curve analyzed the predictive value of combination of FeNO and EO parameters.

ROC curve was used to analyze the predictive value of clinical parameters for *A.f* (+) asthma and ABPA. We found that *A.f*-sIgE, FeNO, SEO%, EO%, and EO had a higher predictive value (cutoff, 4.108; AUC=0.749; CI, 0.629–0.869; cutoff, 55.5; AUC=0.811; CI, 0.707–0.916; cutoff, 6.625; AUC=0.738; CI, 0.619–0.857; cutoff, 8.7; AUC=0.738; CI, 0.619–0.857; cutoff, 0.815; AUC=0.922; CI, 0.856–0.988) than other parameters (**Figures 5A–D**). In differentiating *A.f* (+) asthma from ABPA, the combination of FeNO and EO parameters can optimize the diagnostic efficiency, and the sensitivity and specificity were 80% and 100%, respectively (AUC=0.948; CI, 0.897–0.999) (**Figure 5E**).

**Figure 6:**
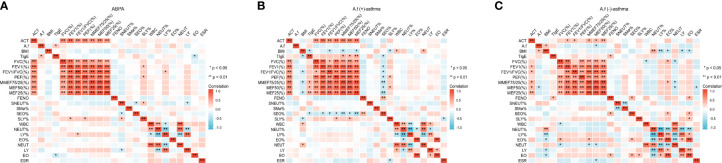
IL-33, IL-25, and TSLP levels in different groups. **(A)** IL-33, IL-25, and TSLP levels in ABPA group; **(B)** IL-33, IL-25, and TSLP levels in *A.f* (+) asthma group; **(C)** IL-33, IL-25, and TSLP levels in *A.f* (−) asthma group. IL-33, interleukin 33; IL-25, interleukin 25; IL-17E, interleukin 17E (can be as IL-25); TSLP, thymic stromal lymphopoietin; ABPA, allergic bronchopulmonary aspergillosis; *A.f* (+) asthma, *Aspergillus fumigatus*-sensitized asthma; *A.f* (−) asthma, non-*Aspergillus fumigatus*-sensitized asthma. *p<0.05 and **p<0.01.

As for the immunological parameters, no significant difference was found with IL-33, IL-17E, and TSLP among these groups (p>0.05) (**Figure 6**).

**Figure 7:**
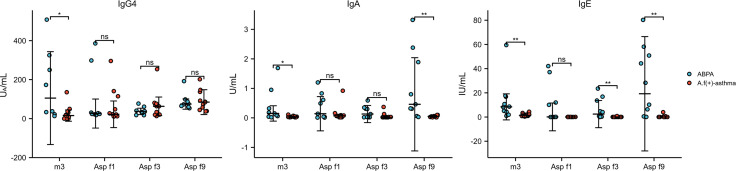
The level of IgG4, IgA, and IgE against *A.f* in different groups. ABPA, allergic bronchopulmonary aspergillosis; *A.f* (+) asthma, *Aspergillus fumigatus*-sensitized asthma; m3, crude extract of A.f; Asp f1, Asp f3, and Asp f9, components of *A.f*; ns, no significance; *p<0.05; **p<0.01.

Compared with specific antibodies and their components between ABPA and *A.f* (+) asthma, the levels of *A.f*-sIgG4, *A.f*-sIgA, and *A.f*-sIgE in ABPA were significantly higher than those in the *A.f* (+) asthma group (105.3 *vs*. 15.6, p=0.023; 0.15 *vs*. 0.03, p=0.014; 8.4 *vs*. 1.44, p=0.002, respectively). sIgA and sIgE components also presented significant differences in IgA of Asp f9 (0.46 *vs*. 0.05, p=0.003) and IgE of Asp f3 (2.43 *vs*. 0.06, p=0.007) and Asp f9 (19.24 *vs*. 0.06, p=0.001) between ABPA and *A.f* (+) asthma (**Figure 7**).

**Figure 8:**
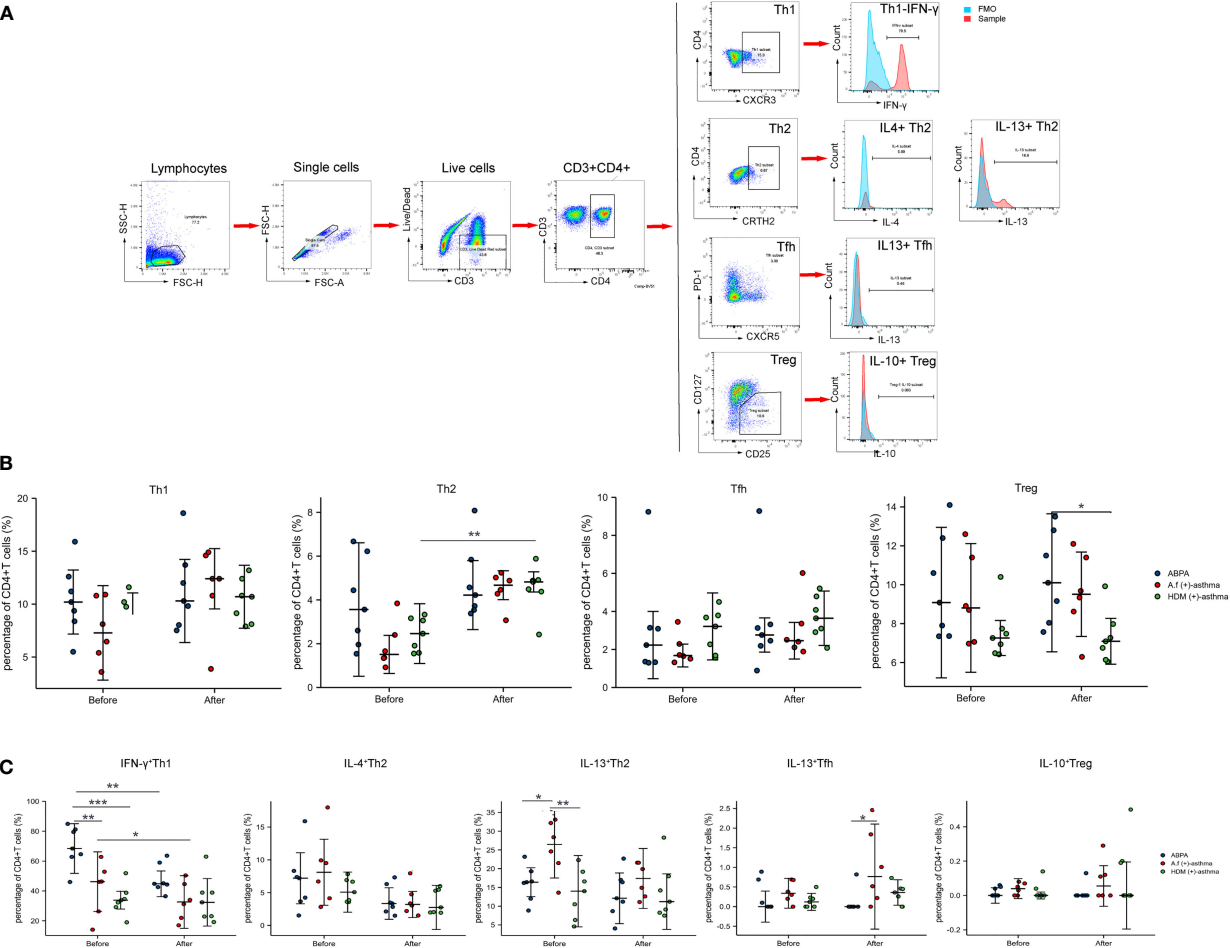
CD4^+^ T-cell subsets and cytokines expression. **(A)** Manual gating strategy for CD4^+^ T-cell subsets and representative plots for Th1 (CD3^+^CD4^+^CXCR3^+^), Th2 (CD3^+^CD4^+^CRTH2^+^), Tfh (CD3^+^CD4^+^CXCR5^+^PD-1^+^), and Treg (CD3^+^CD4^+^CD25^+^CD127^low/−^). IFN-γ^+^Th1, IL-4^+^ Th2, IL-13^+^ Th2, IL-13^+^ Tfh, and IL-10^+^ Treg were representatively exhibited and compared with FMO control. **(B)** The dot plot figures showed CD4^+^ T-cell subsets; **(C)** the cytokines of the Th1 (IFN-γ), Th2 (IL-4 and IL-13), Tfh (IL-13), and Treg (IL-10). ABPA, allergic bronchopulmonary aspergillosis; *A.f* (+) asthma, *Aspergillus fumigatus*-sensitized asthma; HDM (+) asthma, house dust mite-sensitized asthma; Th1, T helper cells 1; Th2, T helper cells 2; Tfh, follicular helper T cells; Treg, regulatory T cells; IFN-γ, interferon γ; IL-4, interleukin 4; IL-13, interleukin 13; IL-10, interleukin 10. *p<0.05, **p<0.01; ***p<0.001.

The PBMCs from seven ABPA patients, six *A.f* (+)-asthma patients, and seven HDM-sensitized asthma [HDM (+)-asthma] patients were collected and exposed to 25 µg/ml *A.f* or HDM extracts, respectively, for 6 h. The percentages of Th1 (CD3^+^CD4^+^CXCR3^+^), Th2 (CD3^+^CD4^+^CRTH2^+^), Tfh (CD3^+^CD4^+^CXCR5^+^PD-1^+^), and Treg (CD3^+^CD4^+^CD25^+^CD127^low/−^) had no significant differences among the three groups. After exposure to allergen, only the Th2 in the HDM group increased significantly (all p<0.05). The signature cytokine-secreting T-cell subsets including Th1 (IFN-γ), Th2 (IL-4 and IL-13), Tfh (IL-13), and Treg (IL-10) were also detected; the percentage of IFN-γ-positive Th1 in the ABPA group was 69.62% ± 13.52%, which was higher than that in the HDM group (34.23% ± 9.95%) (p<0.05). After allergen exposure, the IFN-γ-positive Th1 cells of both ABPA and *A.f* (+)-asthma patients decreased; however, there was no statistical change in the HDM group after HDM exposure (**Figure 8**).

**Figure 9:**
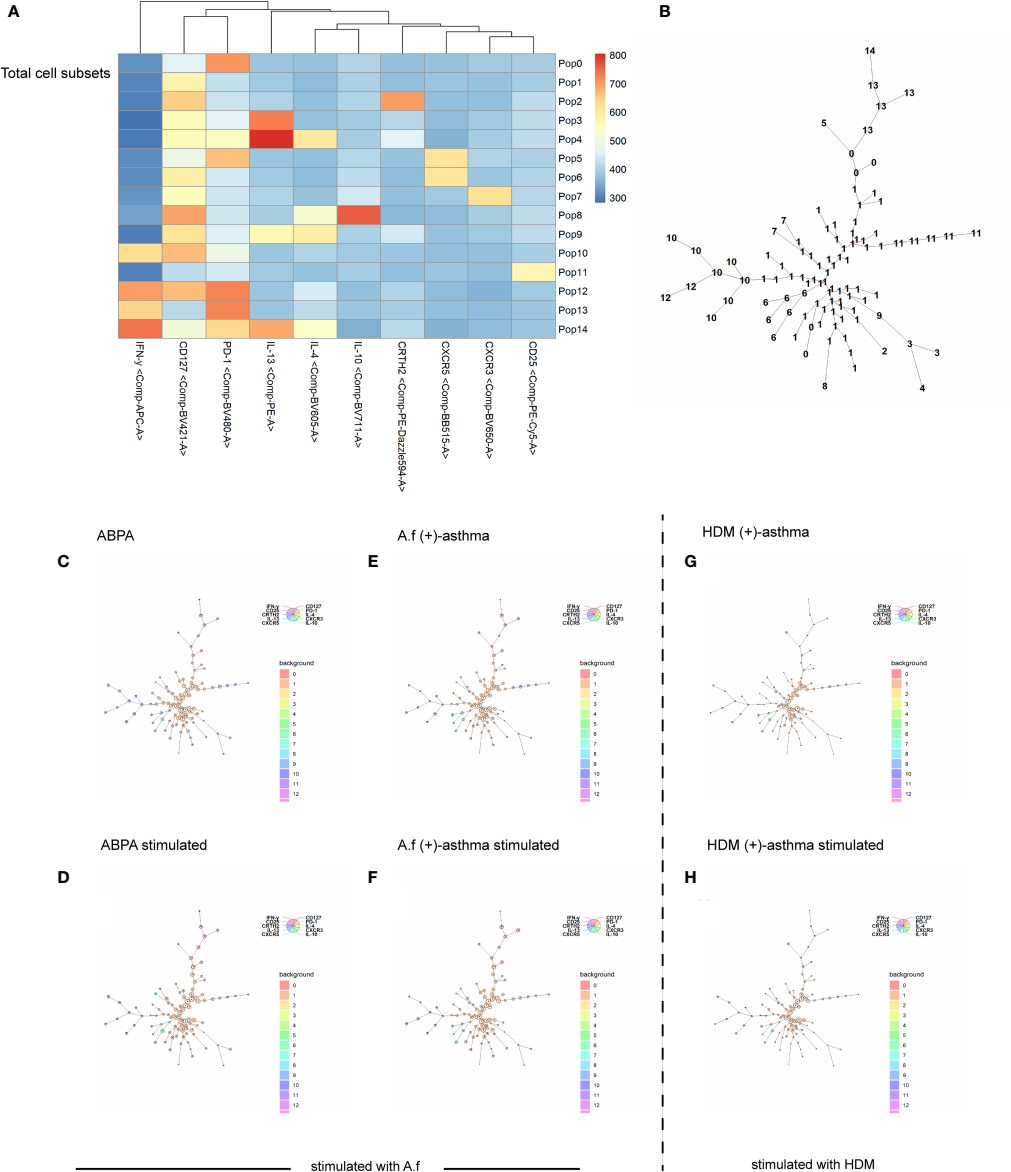
FlowSOM algorithm. Spanning tree visualization of a self-organizing map using compensated flow cytometric data. Data were taken from the lineage (CD3+, CD4+) gate. **(A)** heatmap of the FlowSOM clustering; **(B)** minimal spanning tree for 15 metaclusters; **(C–H)** FlowSOM nodes represent clusters of cells. Metaclusters of the nodes, determined by the map, are represented by the background color of the nodes. *A.f* (+) asthma, *Aspergillus fumigatus*-sensitized asthma; HDM (+) asthma, house dust mite-sensitized asthma.

The FlowSOM algorithm was further used to cluster, visualize, and compare the differences of CD3^+^CD4^+^T cells among the ABPA, *A.f* (+)-asthma patients, and HDM (+)-asthma patients before and after exposure. The result identified that the frequency of metaclusters of pop 10 (CD3^+^CD4^+^PD-1^+^CD127^+^IFN-γ^+^), pop 13 (CD3^+^CD4^+^PD-1^+^IFN-γ^+^) and pop 6 (CD3^+^CD4^+^CD127^+^CXCR5^+^) were higher in the ABPA group compared with that in the *A.f* (+)-asthma group; meanwhile, pop 13 (CD3^+^CD4^+^ PD-1^+^IFN-γ^+^) increased and pop 11 (CD3^+^CD4^+^CD25^+^CD127^+^PD-1^+^) decreased after exposure to allergen in the ABPA patients. The metaclusters of pop 13 also increased in the *A.f* (+)-asthma group after exposure; however, pop 11 was slightly changed by stimulation. For the HDM-allergic patients, the frequencies of pop 3, pop 4, pop 10, pop 11, pop 12, and pop 13 were all lower compared to ABPA group patients (**Figure 9**).

**Figure 10:**
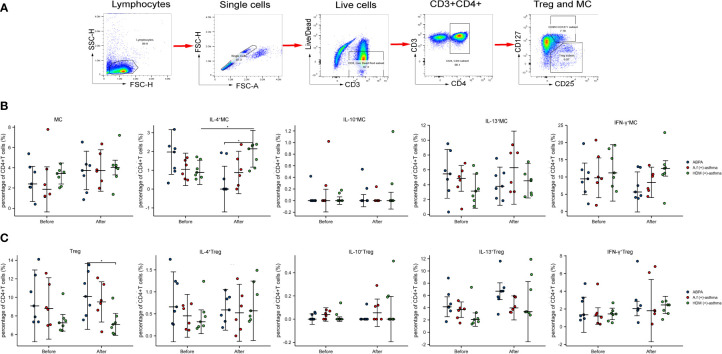
Treg (CD3^+^CD4^+^CD25^+^CD127^low/−^), metacluster (CD3^+^CD4^+^CD25^+^CD127^+^), and their cytokines expression. **(A)** Manual gating strategy for metacluster (CD3^+^CD4^+^CD25^+^CD127^+^); **(B)** the dot plot figures showed the percentage of Treg and the cytokines of the IL-4, IL-10, IL-13, and IFN-γ; **(C)** the percentage and cytokines (IL-4, IL-10, IL-13, and IFN-γ) of metacluster (CD3^+^CD4^+^CD25^+^CD127^+^) are shown. MC, metacluster (CD3^+^CD4^+^CD25^+^CD127^+^); ABPA, allergic bronchopulmonary aspergillosis; *A.f* (+) asthma, *Aspergillus fumigatus*-sensitized asthma; HDM (+) asthma, house dust mite-sensitized asthma; Th1, T helper cells 1; Th2, T helper cells 2; Tfh, follicular helper T cells; Treg, regulatory T cells; IFN-γ, interferon γ; IL-4, interleukin 4; IL-13, interleukin 13; IL-10, interleukin 10. *p<0.05.

We further defined the metacluster of CD3^+^CD4^+^ CD25^+^CD127^+^ cells as the activated Tregs and compared these metacluster cells with Treg (CD3^+^CD4^+^ CD125^+^CD127^low/−^). The metaclusters of the three groups all increased after stimulation [from 2.85% ± 1.62% to 3.49% ± 1.89% in the ABPA group; from 2.95% ± 2.58% to 3.97% ± 1.63% in the *A.f* (+)-asthma group; from 3.20% ± 0.85% to 4.0% ± 1.73% in the HDM (+)-asthma group]. For the ABPA group, the IL-4, IL-13 and IFN-γ^+^ expression on the metacluster all decreased after allergen exposure; however, the expression of these cytokines barely changed in the metacluster of *A.f* (+) asthma, Treg of ABPA, and *A.f* (+) asthma. These cytokines even increased in the metacluster of the HDM (+)-asthma group. The IL-4 expression statistically upregulated in the metacluster in the HDM group after allergen exposure (**Figure 10**). The metacluster of CD3^+^CD4^+^PD-1^+^ also increased in all groups after stimulation; however, only the CD3^+^CD4^+^PD-1^+^IFN-γ^+^ of ABPA decreased (**Supplementary Figure**).

## Discussion

In this study, we compared the clinical and immunological characteristics in ABPA and *A.f*-sensitized asthma patients. We found that the eosinophils in blood and induced sputum and FeNO were significantly higher in ABPA patients compared to *A.f*-sensitized patients. Immunological analysis showed that *A.f*-specific IgE, IgG, IgA, and IFN-γ+Th1 cells also increased in ABPA patients. These findings suggested ABPA patients had more severe eosinophilic inflammation and enhanced Th1 responses compared with *A.f*-sensitized asthma patients. In addition, we first showed that the combination of FeNO and eosinophils parameters had good diagnostic efficiency in differentiating *A.f* (+) asthma from ABPA and thus provided an easy tool other than sIgE and tIgE tests in clinical practice.

The first important thing about this tool is that our study highlighted the importance of eosinophils in the diagnosis of ABPA. Like fungus-sensitized asthma—a type-2 (T2) inflammation caused by eosinophilia (14), ABPA is also a chronic lung inflammation with eosinophilia (14). In fact, eosinophilia has been considered as an important indicator for the diagnosis of ABPA (15). Just as fungal sensitization, whose incidence in patients ranges from 35% to 75% (16), tends to make asthma more severe (17), in our study, we observed that the SEO%, EO, and EO% in induced sputum and peripheral blood of ABPA patients were significantly higher than in *A.f*- and HDM-sensitized asthma patients, which further proved the importance of eosinophils in the diagnosis of ABPA. In addition, we found that in the ABPA group, FeNO levels, which had been regarded as an indicator of eosinophil inflammation in the airway, were significantly higher than those in the other two groups. Our study suggested that ABPA had more severe eosinophilic airway inflammation than *A.f*-sensitized asthma, and FeNO could be considered as a surrogate indicator in the diagnosis of ABPA.

Apart from an emphasis on eosinophilia, we also want to underscore that the measurement of lung function played an important role in the management of asthma and ABPA (18). In the early stage of ABPA, there might be no shadow on lung imaging, but peripheral bronchi and small airway had been involved (19); therefore, it was necessary to assess airway injury with a lung function test. The lung function of ABPA patients was mainly characterized by reversible obstructive ventilatory dysfunction in acute phase, and it was mixed ventilatory dysfunction with reduced diffusion function in chronic phase (20). We found that the lung function parameters decreased in all the three groups, with those in *A.f* (+)-asthma and ABPA groups decreasing more obviously, suggesting that *A.f* might be one of the main reasons leading to accelerated deterioration of lung function. However, there was no statistical difference between the ABPA and the *A.f* (+)-asthma group, which was similar to previous studies (21, 22). Some studies found that bronchiectasis and more severe airflow obstruction were associated with *A.f* sensitization.

Another specific indicator of ABPA was also reviewed in our study. *A.f*-sIgE was considered to be the most sensitive of all laboratory indicators in ABPA diagnosis (21, 22). We analyzed the sensitization of *A.f* in ABPA and *A.f* (+)-asthma groups and found that the level of *A.f*-sIgE in the ABPA group was higher than that in the *A.f* (+)-asthma group. Many studies had shown that the IgE titer of recombinant *A.f* in ABPA patients was higher than that in non-ABPA asthma, which indicated that ABPA patients had a stronger T2 immune response to *A.f* and produce a high level of sIgE and tIgE (23, 24). Thus, the level of *A.f*-sIgE could be used to distinguish ABPA from *A.f*-sensitized asthma; moreover, the cutoff value of *A.f*-sIgE as a diagnostic indicator for ABPA should be elevated at a higher level and investigated in large population rather than the *A.f*-sIgE test result just being positive (above 0.35 KU/L).

Our understanding of ABPA and asthma, despite their similarities in symptoms and treatments, was enhanced not only by our study of the indicators but also by the correlations between these indicators. We used ACT to assess control of *A.f* (+) asthma, *A.f* (−) asthma and ABPA and found that ABPA had the lowest ACT score among the three groups. In the correlation analysis, we also found that ACT was positively correlated with all lung function parameters and negatively correlated with *A.f*-sIgE, FeNO, SEO%, EO%, and EO. Studies had shown that the acute phase of ABPA was associated with a reversible decline in lung function (25), and eosinophils were the primary mediators of inflammatory activity in ABPA (26), which was consistent with our findings that ABPA had the worst control level, which may be accounted for by the decreased lung function and eosinophilia. In addition, we found a negative correlation between lung function parameters and SEO% in the three groups; however, no correlation between peripheral blood eosinophils and lung function parameters was observed in the ABPA group, which suggested that sputum eosinophils were more sensitive in reflecting exacerbation of lung function in ABPA than blood eosinophils.

Nitric oxide (NO) mediated a variety of physiological reactions at low level, while a high level of NO was involved in the occurrence of innate immunity and chronic inflammatory diseases (27). In our study, there was a significant negative correlation between ACT score and FeNO in the three groups, which indicated that patients with higher FeNO tend to suffer from uncontrolled asthma or ABPA. It is suggested that FeNO could be a biomarker that integrates both airway inflammation and lung function changes (28), which was consistent with our findings that a negative correlation existed between FeNO and lung function parameters. We also found that *A.f*-sIgE was positively correlated with FeNO, SEO%, EO%, and EO and negatively correlated with ACT and all lung function parameters, which re-confirmed the relationship of sensitization of *A.f* and poor control of asthma and ABPA.

To distinguish *A.f* (+)-asthma patients from ABPA accurately, we further compared the ROC curves of the potential clinical indicators. Our study suggested that some parameters such as *A.f*-sIgE, FeNO, SEO%, EO%, and EO had good diagnostic efficiency. However, the cutoff values of these indicators were different from the current criteria. For example, the current criteria proposed that *A.f*-sIgE is >0.35 KAU/L and EO >0.5×10^9^/L for the diagnosis of ABPA (11), while we found that the best diagnostic efficiency could be achieved when the cutoff value of *A.f*-sIgE was 4.108 KU/L and EO was 0.815×10^9^/L. We strongly suggest that the optimal cutoff values of these indicators should be investigated and validated in a large population. We also tried to incorporate the indicators into the ROC analysis. Just as we expected, a sensitivity of 80% and a specificity of 100% were obtained in the combined FeNO and EO diagnostic model, which implied that for *A.f*(+)-asthma and ABPA with high-level tIgE, the combination of FeNO and EO for differential diagnosis was reliable and had the advantages of convenience and low cost.

We also studied epithelial-cell-derived IL-33, IL-25, and TSLP, which were regarded as alarmins and played pivotal roles in the initiation of allergic inflammation in asthma (29). Their receptors widely expressed in structural cells and innate and adaptive immune cells, contributing to the airway disease by driving inflammatory processes (30). The expression of IL-33, IL-25, and TSLP should be higher in ABPA and asthma theoretically. However, we did not find a significant difference in serum IL-33, IL-25, and TSLP among ABPA, *A.f*-sensitized asthma, and non-*A.f*-sensitized asthma, which was consistent with previous studies (26). The explanation for the absence of increased IL-33, IL-25, and TSLP may be that we examined these cytokines in serum samples rather than in bronchial epithelium. It is possible that these cytokines in the sputum will provide more favorable information. However, lack of enough sputum samples did not allow us further pursuit, which was a limitation of our study. Simultaneously measuring the cytokine secretion and mRNA expression of the epithelium cells may provide robust evidence for roles of these cytokines in ABPA and *A.f*-sensitized asthma.

Genetic factors and activation of bronchial epithelial cells in asthma or cystic fibrosis are responsible for CD4^+^Th2 lymphocyte activation and production of A.f-sIgE, A.f-sIgG and A.f-sIgA (31). ABPA is a disease commonly associated with asthma and cystic fibrosis; theoretically, specific antibodies to *A.f* in ABPA would be higher. In our study, we found that sIgG4, sIgA, and sIgE in ABPA were higher than those in *A.f*-sensitized asthma, which coincided with the previous study (32). Similarly, one study that looked into the total IgG4 level (not *A.f*-IgG4) also found that IgG4 levels in ABPA patients were higher than that in asthma (33). Apart from the *A.f*-specific antibodies analysis, we further investigated the *A.f* component antibodies profiles in the ABPA and *A.f*-sensitized asthma patients. Asp f1 was the major component of *A.f*, and Asp f3 and Asp f9 were components that often caused cross-sensitization. In our study, we that found Asp f9-sIgA and Asp f9-sIgE and Asp f3-sIgE in the ABPA group was significantly higher than those in *A.f*-sensitized asthma, however, specific antibodies (including IgG4, IgE, and IgA) against Asp f1 showed no difference in the two groups. Our study was not consistent with previous studies that suggested that sIgE against Asp f 1, Asp f 2, Asp f 4, and Asp f 6 in ABPA were higher than that in *A.f*-sensitized asthma (34). This discordance may be attributed to small sample sizes in the relevant studies. In addition, the clinical significance of specific antibody levels of Asp f3 and Asp f9 between the two groups remains unclear. Our study provided preliminary information of specific antibodies against Asp f3 and Asp f9 in ABPA and *A.f*-sensitized asthma and the detailed role of these antibodies need to be elucidated in further studies.

ABPA- and *A.f*-sensitized asthmas are regarded as type 2 inflammation, and the activation of Th2 cells play important roles in their pathogenesis. Becker et al. reported that A.f plays a primary role in the induction of a Th2 response in human PBMCs (35). Emerging data have supported that APBA pathophysiology shifted from immune deviation toward favored Th2 responses (36). However, A.f is an invasive fungus and could also elicit type 1 inflammation by pathogen-associated molecular patterns (PAMPs) in which innate immune cells and antigen presenting cells were involved (37). In our study, we found that the IFN-γ^+^Th1 cells especially CD3^+^CD4^+^PD-1^+^CD127^+^IFN-γ^+^T cells significantly increased in ABPA patients when compared to A.f-sensitized patients, which suggested that there might be more severe airway epithelial damage in the ABPA patients, driving a shift to Th1 inflammation. However, we did not find the difference in epithelial damage-related cytokines such as IL-25, IL-33, and TSLP in sera of the two groups. As we mentioned above, we think the cytokine analysis that directly targeted epithelial cells will provide more details of mucus barriers dysfunction in ABPA. The FlowSOM algorithm confirmed that the frequency of CD3^+^CD4^+^PD-1^+^CD127^+^IFN-γ^+^T cells was higher in ABPA patients. It was reported that PD-1 maintains tolerance and CD127 could be upregulated in activated Treg cells (38, 39). We further gated metacluster (CD3^+^CD4^+^ CD25^+^ CD127^+^) and metacluster (CD3^+^CD4^+^ PD-1^+^), the two metaclusters increased in all the groups after stimulation. However, cytokines, especially the IFN-γ of the two metaclusters declined only in ABPA, although no statistical significance was found. The decline in the cytokine expression in ABPA patients might impair the tolerance function of the cells and lead to disease progression. However, more samples are needed to confirm our findings.

In conclusion, *A.f* sensitization was an important factor leading to the decline of lung function and the worsening of asthma control in asthma and ABPA patients. ABPA patients showed a higher FeNO level and eosinophils in blood and induced sputum, suggesting a more severe eosinophilic inflammation in ABPA pathogenesis. The combined application of FeNO and EO was reliable and convenient for the differential diagnosis of ABPA and *A.f*-sensitized asthma. These findings help to discriminate ABPA from *A.f*-sensitized patients, and the indicators should be further validated in large population.

## Data availability statement

The raw data supporting the conclusions of this article will be made available by the authors, without undue reservation.

## Ethics statement

This study was reviewed and approved by Independent Ethical Committee of First Affiliated Hospital of Guangzhou Medical University and Tongji Hospital, Tongji Medical College, Huazhong University of Science and Technology. Written informed consent to participate in this study was provided by the participants’ legal guardian/next of kin.

## Author contributions

RZ and HC conceived and designed the project. HC was responsible for data analysis and wrote the first draft of the manuscript. RZ revised the raw manuscript. XZ and NA were responsible for patient’s enrollment and data analysis. YY and DM collected clinical data. LY and LZ were responsible for allergen components tests and FCM. QJ contributed to IgE test by ImmunoCAP. All authors contributed to the article and approved the submitted version.

## Acknowledgments

We express our gratitude to Hangzhou Zheda Dixun Biological Gene Engineering Co, LTD for the kindly providing *A.f* extract and components of sIgE, sIgA, and sIgG4 test kits. We also thank ALK (Horsholm, Denmark) for supplying HDM extract.

## Conflict of interest

The authors declare that the research was conducted in the absence of any commercial or financial relationships that could be construed as a potential conflict of interest.

The reviewer PZ declared a shared affiliation with the authors XZ, NA to the handling editor at time of review.

## Publisher’s note

All claims expressed in this article are solely those of the authors and do not necessarily represent those of their affiliated organizations, or those of the publisher, the editors and the reviewers. Any product that may be evaluated in this article, or claim that may be made by its manufacturer, is not guaranteed or endorsed by the publisher.

## Supplementary material

Supplementary Figure The percentage and cytokines (IL-13 and IFN-γ) of metacluster (CD3^+^CD4^+^PD-1^+^). MC: metacluster (CD3^+^CD4^+^PD-1^+^); ABPA: allergic bronchopulmonary aspergillosis; A.f (+) asthma: aspergillus fumigatus sensitized asthma; HDM (+) asthma: house dust mite sensitized asthma; IFN-γ: Interferon γ; IL-13: interleukin 13. * was P<0.05.
