# Supplementary figures and images for: Clinical and immunological characteristics of Aspergillus fumigatus-sensitized asthma and allergic bronchopulmonary aspergillosis
Source: Front Immunol. 2022 Aug 2;13:939127. doi: 10.3389/fimmu.2022.939127 (PMC9379317; doi:10.3389/fimmu.2022.939127)

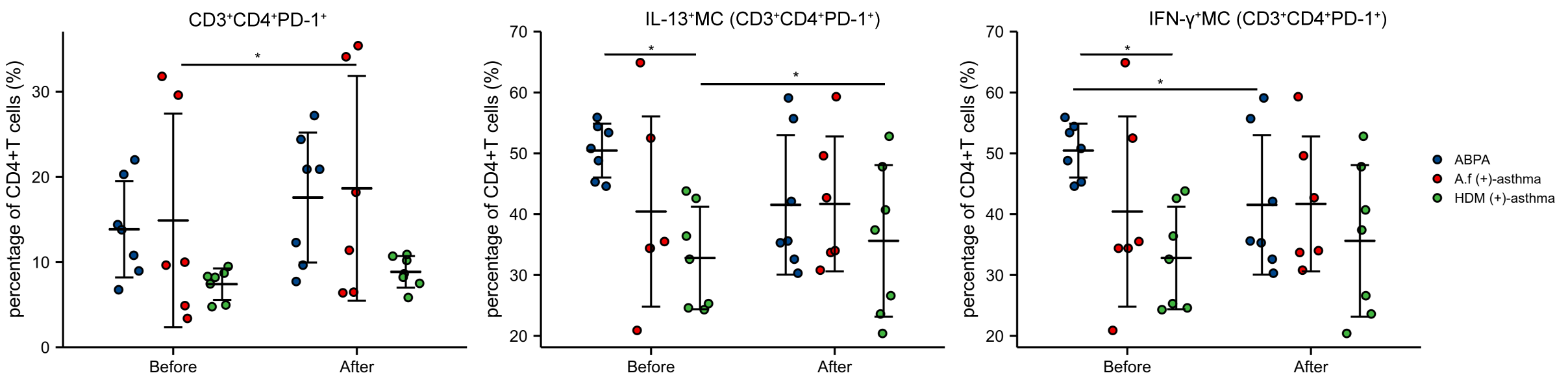

Supplement: Supplementary Figure — The percentage and cytokines (IL-13 and IFN-γ) of metacluster (CD3+CD4+PD-1+). MC: metacluster (CD3+CD4+PD-1+); ABPA: allergic bronchopulmonary aspergillosis; A.f (+) asthma: aspergillus fumigatus sensitized asthma; HDM (+) asthma: house dust mite sensitized asthma; IFN-γ: Interferon γ; IL-13: interleukin 13. * was P<0.05. [file Image_1.tif]
